# When Leadership Drives Nurses Away: Empirical Research Qualitative on High Turnover Rates Reasons

**DOI:** 10.1002/nop2.70271

**Published:** 2025-07-08

**Authors:** Saleem Al‐Rjoub

**Affiliations:** ^1^ Faculty of Nursing, Department of the Community and Mental Health Nursing The Hashemite University Zarqa Jordan

**Keywords:** leadership, nurse leader, registered nurse, turnover

## Abstract

**Aim:**

This study aimed to investigate the reasons that affect nurses' decisions to leave their setting. Nurse turnover impacts organisational costs, organisational stability, and quality of care. The role of nurse leaders' practices in nurse turnover remains underexplored.

**Design:**

Descriptive phenomenological design was used in the current study.

**Method:**

Giorgi's phenomenological method was used to explore the staff nurses' lived experiences and perceptions of turnover. The data was collected from semi‐structured interviews with nurses who had left their current positions, moved to another organisation, changed professions, or retired.

**Results:**

Five themes emerged from the analysis: (1) poor communication, (2) a lack of leader support, (3) workloads and staffing issues, (4) a lack of professional development opportunities, and (5) inconsistent policies and leadership.

**Conclusion:**

Nurse leaders and administration play a critical role in driving nurses to stay or to leave the healthcare organisation by providing different and important practices and behaviours. Reducing the nursing turnover rate can create a more stable environment and enhance nurse satisfaction, which leads to better patient care.

**Implications for Nursing Administration:**

Nursing administration must create supportive workplace environments by enhancing leadership practices to reduce the nursing turnover rates, especially among new graduate nurses.

**Impact:**

The current study addresses the nurse turnover issue, with a particular focus on the role of nurse leaders' practices in nurse turnover. High nursing turnover rates impact organisational costs, organisational stability, and quality of care. This study will have a significant impact on nursing leadership, healthcare administration and policymakers.

**Patient or Public Contribution:**

No Patient or Public Contribution.

## 1 Introduction

The nursing profession, a keystone in healthcare organisations, is critical to ensuring healthcare quality and patient outcomes. However, nurses have a significant problem due to high turnover rates. Nurse turnover is defined as the rate at which nurses leave their current positions, move to another organisation, change professions, or retire (Hu et al. 2022). Nursing turnover leads to the fragmentation of patient care, disrupts team cohesion and increases expenses for healthcare organisations, including recruitment and training costs during transition periods (Andrews et al. 2024; Church et al. 2023). These transition periods can also adversely affect patient satisfaction, patient care, organisational stability and additional stress on remaining staff members (Guixia and Hui 2020; Randa and Phale 2023). Moreover, the loss of highly experienced nurses can impact negatively on the quality of patient care, as newly hired nurses may not be as familiar with patient needs or organisational procedures (Randa and Phale 2023). Additionally, the remaining nurses may experience increased stress and workloads, which lead to increased turnover rates.

The healthcare sector is characterised as a dynamic development sector that is marked by modifications in healthcare policies, changes in patient needs, and developments in technology. Nursing turnover is one of the dynamic issues in the healthcare sector around the world. Healthcare institutions need to create a healthy workplace setting for nurses to reduce the turnover rate by providing opportunities for professional development and fostering positive leadership practices. This will create a more stable and efficient healthcare environment to meet the patient's needs (Vardaman et al. 2020). Healthcare organisations can improve nurse retention, enhance the quality of patient care, and guarantee the sustainability and stability of the nursing workforce when determining the reasons for nurse turnover. Determining the primary reasons behind nurse turnover is essential for developing strategies to reduce the chance of nurses quitting their positions. These strategies require a comprehensive approach and ongoing studies to determine how these reasons influence nurses' decisions to leave.

## 2 Background of the Study

Extensive studies have investigated several reasons that impact nurse turnover. These reasons included workload, organisational climate, interpersonal relationships, job dissatisfaction, burnout and leadership styles (Cho et al. 2022; Lee et al. 2020; Liu et al. 2023; Magbity et al. 2020; Phillips 2020). Workload refers to the amount of care and time that one staff nurse spends on patient care, workplace tasks, and professional development (Alghamdi 2016; Phillips 2020). Handling the workload can lead to high care quality and job satisfaction, as well as reduced turnover rates.

Organisational climate is the culture and overall environment inside the organisation. A healthy organisational climate is characterised by effective communication, collaboration, and mutual respect. This climate can reduce turnover and enhance job satisfaction (Laschinger et al. 2012). Interpersonal relationships, particularly with colleagues and supervisors, are critical for reducing turnover rates (Liu et al. 2023). Negative relationships can lead to stress and burnout, while supportive relationships can enhance job satisfaction and increase retention rates.

Another reason contributing to nurse turnover is job dissatisfaction. Studies indicate that dissatisfaction with the work environment, organisational support and workload leads to increased turnover rates (Lee et al. 2020). Burnout is associated with increased intentions to leave their positions. Burnout is a prolonged response to chronic stressors on the job. Maslach et al. (2001) determined burnout to be characterised by depersonalization, emotional exhaustion and reduced personal accomplishment. High levels of burnout are also closely linked to nurse turnover (Jun et al. 2021).

As previously mentioned, various reasons influence nurse turnover; however, the role of nurse leaders is particularly significant. Nurse leaders' styles and behaviours directly affect job satisfaction, the work environment and the retention of nursing staff (Cho et al. 2022). The autocratic leadership style has been shown to increase turnover rates due to poor communication, centralised decision‐making, a lack of trust and support, and insufficient guidance (Magbity et al. 2020). Conversely, transformational leadership is characterised by motivating and inspiring nurses, which is significantly associated with reduced turnover rates among nurses (Magbity et al. 2020). Under the transformational style, nurses foster professional development, engage with their team members and create a sense of purpose and community.

The COVID‐19 pandemic impacts nursing staff turnover, workload, and job satisfaction. According to a systematic review by Yaraghi et al. (2022), leadership plays a critical role in addressing the challenges faced by healthcare workers during the pandemic. Leadership can create a supportive workplace environment that provides emotional and logistical support, which leads to fostering job satisfaction and reducing turnover intentions among nurses. The study also highlighted significant financial strains, which were unable to offer adequate financial incentives to retain staff. These financial constraints combined with increased workloads contributed to the limited ability to offer competitive wages or bonuses. These constraints suggest that healthcare leaders must adopt a holistic approach to reduce staff turnover, focusing not only on financial compensation but also on fostering a supportive work environment that addresses the psychological and emotional well‐being of staff.

Negative and abusive leadership behaviours include a lack of empathy, lack of support, lack of respect, poor communication, selfishness, and favouritism, which contribute to job dissatisfaction and increased turnover intentions (Lavoie‐Tremblay et al. 2016). On the other hand, healthy leadership behaviours include encouraging teamwork, fostering professional development opportunities, acknowledging achievements, offering adequate resources, encouraging to give suggestions and feedback, and focusing on two‐way communications which can lead to reducing turnover rates and enhancing job satisfaction among nurses (Cziraki et al. 2020). This study used a descriptive phenomenological approach to identify the reasons for nursing turnover.

## 3 Methodology

### 3.1 Research Design

This study used a descriptive phenomenological design. The purpose of this design is to discover the perceptions and lived experiences of nurses regarding the role of nurse leaders in turnover. Descriptive phenomenology allows researchers to collect nuanced, rich data that describe an in‐depth understanding of nurses' decisions to leave their positions. Additionally, this design emphasises recognising the study phenomena from the participants' experiences and perspectives (Giorgi 2012). This design also leads to reconsidering new practices and policies in a relevant field (Creswell and Poth 2016).

### 3.2 Sample

After getting approval from The Hashemite University's IRB, purposive sampling was used to recruit 20 nurses who left their current positions, either moved to another organisation, changed professions, or retired. This study was conducted in Jordan and focused on registered nurses who were employed in different healthcare facilities. Participants were recruited via phone calls and email from their professional and social networks. Sixteen nurses were interested in participating in the study who had left their current positions, moved to different healthcare settings, changed professions, or retired. However, 4 of the 16 nurses who did not meet the eligibility criteria were excluded from participation. Twelve nurses were eligible and participated in the study. Criteria for study inclusion consisted of RNs who have left their positions; nurses from various specialties; nurses with different years of experience, from newly graduated to highly experienced; nurses from different hospital types; and able to speak English. Exclusion criteria were non‐registered nurses and nurses who worked non‐bedside and who were not involved with patient care.

### 3.3 Instruments

Each participant has been asked to complete a short demographics questionnaire. Then, complete a 45‐min audio‐recorded individual interview. Based on the author's experiences, interview questions have been developed. The following central question guided this study: ‘How do nurse leaders' practices and behaviors impact your decision to leave your positions in healthcare settings?’. Moreover, Table 1 includes several questions that guide the interview to gain valuable insights into the phenomenon.

**TABLE 1:** List of interview guide questions.

How do nurse leaders' practices and behaviours impact your decision to leave your positions in healthcare settings?
Tell me if you feel that your nurse leader was not sensitive to your personal needs and requirements.
What were the main reasons affecting your decision to leave your job?
What were the leaders' practices or behaviours that impacted your decision to leave your job?

### 3.4 Data Collection

The data was collected from 12 registered nurses using semi‐structured interviews. The participants were interviewed face‐to‐face in a private place or through private calls to encourage participants to speak up freely. To ensure participants' confidentiality, the interviewer assigned a specific number for each participant. Each interview lasted 45 min. Four researchers participated in the interview process. After receiving consent forms from each participant, the study goals were reviewed with the participants. The interviews were audio‐recorded with the participants' agreement. Audio tapes were erased after completing the study.

Similar guided questions were asked for each participant (Table 1). These questions focused on reasons affecting their decision to leave their jobs, nurses' experiences with their leaders, and the impact of leaders' practices and behaviours on their decisions to leave their jobs. Each interview provides explanations, enlightens, and specifies information regarding the participant's meaning of a lived experience related to nursing turnover. The data collection process was conducted for 2 months, from December 23, 2022, to January 23, 2023.

### 3.5 Data Analysis

This study uses a qualitative research design. The data analysis followed Giorgi's phenomenological method (Giorgi 2012). This method involves four steps (Table 2). After following these steps, a general structure of the nurses' experience with their leaders and a comprehensive understanding of the nurses' lived experiences regarding turnover is presented. Then, the keywords and key themes were identified. To ensure the trustworthiness of the findings, triangulation was used by comparing the findings from different participants and cross‐referencing the themes identified across multiple interviews. This helped to prove that the identified themes were applicable and reliable to the experiences of a group of nurses (Heale and Forbes 2013).

**TABLE 2:** Giorgi's phenomenological method steps.

*Giorgi's phenomenological method steps*
Reading the entire description to obtain a sense of the whole: Comprehensive reading of each interview transcript
2 Generating meaning units: Systematically broke down the transcribed interviews into units. Each unit represented distinct experiences, feelings or thoughts
3 Transitioning the meaning units into a psychological perspective: Involves transforming meaning units into phenomenological descriptions by interpreting each unit with the participants' lived experience and ensuring that each participant's experience was captured
4 Synthesising these perspectives to describe the essence of the study phenomenon: Synthesising the transformed descriptions into a unified structure that captures the core themes emerging from the data

### 3.6 Ethical Considerations

The study was approved by the Institutional Review Board (IRB) of ‘The Hashemite University’ University on November 19, 2023 (No: 26‐1‐2023‐2024). Informed consent was gained from all participants. The confidentiality of all participants is assured by assigning a number for each participant, which was used to analyse and collect the findings. All interviews were conducted through private calls or in a private place away from their workplace to address potential power imbalances between participants and their nurse leaders. Participants were informed that they could withdraw from the study at any time without any negative consequences with their employer.

## 4 Findings

### 4.1 Characteristics of Participants

The study included 12 Registered nurses from different hospital types: governmental, private, teaching and military. 75% were male and most participants (60%) were less than 25 years old. Most participants were new graduate nurses with less than 3 years of experience as registered nurses. More participants' characteristics are found in Table 3.

**TABLE 3:** Participant demographic characteristics.

Level	Value	
	*N* = 12	%
Gender		
Female	3	25
Male	9	75
Age in years		
Less than 25	10	83
25–35	2	17
35–45	0	00
45–55	0	00
55 and older	0	00
Number of years as a clinical RN		
Less than 1 year	6	50
1–2	4	34
3–4	1	8
5–9	1	8
More than 10 years	0	00
Employment		
Unemployed	1	8
Employed	11	92
Retired	0	00
Type of the hospital		
Governmental hospital	3	25
Private hospital	3	25
Military hospital	3	25
Teaching hospital	3	25
Ward or unit average daily census		
1–5 patients	0	00
6–10 patients	0	00
11–15 patients	2	17
16–20 patients	4	34
More than 20 patients	6	50
Ward or unit's organisational structure		
Vertical	9	75
Horizontal	0	00
Matrix	0	00
Unclear	3	25

### 4.2 Thematic Analysis

Five major themes emerged that contributed to the reasons causing a high turnover rate among nurses. Each theme highlights the different practices and behaviours of nurse leaders' effect on nurses' decisions to leave their work setting. These themes included poor communication, a lack of leader support, workloads and staffing issues, a lack of professional development opportunities, and inconsistent policies and leadership. Sample participants' statements are included to illustrate each theme. Table 4 shows the key themes that were identified from the analysis, accompanied by illustrative quotations.

**TABLE 4:** Themes and sample participants' statements.

Themes	Sample participants' statements
**Poor communication**	‘Oh yeah, I found myself feeling lost because of poor communication from my supervisor. It takes many times, sometimes weeks when I would hear about any changes to policy or procedure, but…my manager never informed me directly. She left me confused and unsure of what I was supposed to do.’ (Participant 11) ‘The communication was just awful in my unit. Always, my colleagues tell me any important updates on procedures rather than hear them from my manager. It looks like I was left in the dark about what was going on inside my department, and at this moment, I couldn't trust that this hospital had my best interests in mind.’ (Participant 5)
**Lack of leader support**	‘…No matter how much I worked, umm extra shifts, heavy workloads or taking on challenging cases, I felt like my efforts were completely ignored. Honestly, I might never hear the “thanks” word come from my manager's mouth’ (Participant 2) ‘Well! There was no support from my boss during my shifts and I felt completely on my own, especially when things got tough. I just have no motivation to work more…I just felt demoralised’ (Participant 12)
**Workloads and staffing issues**	‘The workload was just overwhelming and frustrating. My manager seemed like she didn't care about the staffing issues. Some shifts felt like I was doing the job of tens of patients, and I couldn't keep up anymore.’ (Participant 11) ‘I was running like a marathon in every shift. There was no appreciation for how hard we were working to cover all tasks.’ (Participant 10)
**Lack of professional development opportunities**	‘When I started my job, I hoped there would be room to develop myself and my career. I wanted to have special training in some procedure and continue my graduate studies but…there was nothing like that. I didn't feel that there would be any professional growth’ (Participant 6) ‘I came in hoping that I could get some kind of support to continue my graduate education, a master's degree. However, after I started my job in the hospital, my colleagues told me that no one was offering any assistance for further graduate studies…I felt like I was stuck.’ (Participant 12)
**Inconsistent policies and leadership**	‘For my first 5 months working at my department, I had worked with 2 new managers and every manager had a different approach and style. If the first manager would say something, the second manager would change it and say new things. Always I was confused…I never knew what was expected anymore.’ (Participant 3) ‘My new manager suddenly imposed new policies without any prior discussion with us. I was shocked when I was told to follow new rules that had never been communicated before…I felt unsupported.’ (Participant 1)

#### 4.2.1 Poor Communication

In this study, unclear or ineffective communication between leaders and staff nurses was a significant theme identified throughout the data collection process. Poor communication with leaders made a large contribution to nurses' decisions to leave their positions and seek another setting. A participant explained that unclear communication could cause frustration and confusion while performing their daily tasks.

Unclear communication from my manager was incredibly frustrating. I frequently had no idea about new or even changes in a procedure. It made my job confused and difficult. (Participant 11)

#### 4.2.2 Lack of Leader Support

Throughout the development of themes, it was determined that lack of leader support and recognition led to the feeling of being unappreciated and undervalued. Data revealed that lack of nurse leader support was a major reason for the staff's decision to leave their setting. Eight participants contributed their experience with this issue. Participants described their daily hard work without nurse leader support and their feelings.

I felt ignored. No matter how many extra shifts or how hard I worked. My hard work was never acknowledged by my superior. I feel like I was only such a cog in a machine. (Participant 2)

Another participant explained that a lack of nurse support could cause demoralisation.

I remember that I had the most challenging times in my shift, there was no support from the management. It was demoralizing. (Participant 8)

#### 4.2.3 Workloads and Staffing Issues

The study's participants mentioned that heavy workloads lead to feelings of high stress and fatigue. The qualitative data suggests that nurse leaders who do not address proper staffing shortages can contribute to nurse turnover. One nurse mentioned the reason for leaving the hospital by stating:

We always have short‐staffed in my unit, and my supervisor didn't seem to care about it. The biggest problem was that my supervisor did not distribute workloads fairly. Some staff had a large amount of work and tasks and others did not have any assignments. When I asked my supervisor why this happened. She said that's not your business. That was the main reason to left my job. (Participant 9)

#### 4.2.4 Lack of Professional Development Opportunities

Limited opportunities for professional growth were identified in several of the interviews that also emerged as contributing to fostering nurse turnover. A newly graduated nurse was looking forward to changing his hospital because there was no room for his career advancement, which led him to a sense of stagnation and frustration, stating:

In my old hospital, there was no room for advancement or growth. There was no opportunity offered by the management. It felt like a dead‐end job. (Participant 6)

#### 4.2.5 Inconsistent Policies and Leadership

Participants expressed that frequent changes in policies or leadership without follow‐up and proper implementation were contributors to feelings of dissatisfaction. The qualitative data revealed that unplanned changes in policy or leadership contributed to the difficulty of creating a stable working environment.

Every few months, my department seemed to have a new manager with a different set of rules. This change made it impossible to work in a stable working environment. This made me dissatisfied and made me think if I wanted to change my job. Then that was what happened. I never feel a stable in working environment. (Participant 3)

A participant was evaluated by new measures not previously discussed because of hiring a new manager who made these new measures without any notification.

So, it was odd. Why this was coming to me? When talking to the chief nursing officer, she said that they had created new measures from a new administrator to go ahead and send those applications to me. At this point, we were surprised. And I felt like they violated the ethics. (Participant 1)

## 5 Discussion

This study's themes reflect different ways in which negative leadership practices can contribute to nurses' decisions to leave their positions. Addressing these practices can help create a healthy working environment and reduce the nurse turnover rate. High turnover rates affect the financial stability of healthcare organisations, the quality of patient care, and the overall morale of nursing staff (Andrews et al. 2024; Church et al. 2023). The findings of this study reveal five predominant themes: poor communication, lack of leader support, unreasonable workloads and staffing issues, lack of professional growth opportunities, and inconsistent leadership and policies. Each of these themes highlights the essential role that nurse leaders play in either reducing or increasing turnover rates.

One of the most pressing concerns is the negative effect of perceived limited support from nurse leaders. The study's participants consistently reported feelings of being invisible, neglected, unappreciated and undervalued, which made eight out of 12 nurses seek employment elsewhere. Effective and clear communication is a keystone of nurse leaders within healthcare settings (Fowler 2023). Lack of leader support not only impacts job satisfaction but also increases the likelihood of turnover and burnout (Grossman 2021; Hult et al. 2023). Many of the interviews highlighted several instances where poor communication from nurse leaders caused mistakes, confusion, and feelings of frustration among nurses. Unclear communication about changes, procedures, and policies created an environment of uncertainty within the healthcare setting. Participants experienced a lack of trust in their leaders when they felt left in the dark about essential procedures or policy updates. This unclarity in communication can lead to an extra workload for nurses as they are challenged to manage unclear job expectations, adjust to unexpected changes, and clarify tasks (Fowler 2023). Moreover, poor communication may exacerbate workload issues at the healthcare organisation by reducing the timely allocation of resources or constraining adequate support (Guixia and Hui 2020). The literature confirmed that improving communication strategies could significantly reduce turnover rates (Duru and Hammoud 2022).

Many nurses reported feelings of instability with frequent changes in policies and nurse leaders. These changes create a difficult work environment to provide reliable patient care and establish consistent daily operations. Inconsistency in expectations and rules not only disrupts routines but also damages nurses' trust and confidence in their supervisors (Wang et al. 2022). Stability and continuity in leadership are required to be addressed in healthcare settings to ensure that changes are properly implemented and well communicated.

This study also found that the limited opportunities for career advancement and professional development are concerns raised by participants. Many new graduate nurses expressed disappointment in limited opportunities for continuous academic degrees such as a master's degree, further training programs, specialisation and promotion. These limitations influence feelings of frustration and a lack of motivation to stay in the healthcare sector. Most nurses reported that healthcare organisation's management often failed to offer the necessary encouragement and support for their staff needs. This leads to reduced job satisfaction, job stagnation, burnout, and higher turnover rates (Shi et al. 2015; Waltz et al. 2020; Wei et al. 2020). Moreover, the participants expressed dissatisfaction with potential misalignment between the realities of their new role and their expectations at hiring. New nurses may expect career growth immediately after hiring, including certifications, further education or specialty training, which are usually unavailable immediately after hiring. This could lead to a sense of stagnation and frustration.

The final theme centers on chronic understaffing and workloads. Nurses consistently reported feeling their concerns and requests for additional staffing were ignored by their organisation's management. The literature confirmed that excessive workload leads to emotional and physical exhaustion among nurses, which not only places high pressure on nurses but also compromises the quality of patient care (Peng et al. 2023; Subramony et al. 2024). The theme of chronic understaffing and workloads interacts with lack of leader support. Nurses who report a lack of leader support face challenges in managing heavy workloads, exacerbating stress and frustration. Also, inadequate staffing is interrelated with feelings of neglect and disempowerment when leadership does not provide necessary resources or address workload concerns. Lack of the leader's support during times of heavy workloads not only affects the performance of the nurse but also damages team dynamics. Nurses may become stressed, dissatisfied, and less motivated if they feel their feedback is ignored. Therefore, effective leadership is critical in actively managing workloads and fostering a supportive environment. Leader's support has positive effects on decreased workloads, improved job satisfaction and reduced turnover rates.

Leadership provides valuable insights into reducing nurse turnover rates. Effective nurse leader creates an empowering work environment by promoting open communication, supporting and recognising staff contributions, ensuring fairness and equity, providing professional development opportunities, and stabilising leadership and concertizing policies (Cziraki et al. 2020). This can significantly enhance nurses' retention and job satisfaction. Highlighting the potential reasons that cause an increase in nurse turnover rates can help healthcare settings develop targeted interventions to maintain a stable healthcare system and retain their nursing workforce.

### 5.1 Strengths, Limitations and Recommendations for Further Research

A qualitative method provides rich, deep insights into the perceptions and experiences of staff nurses regarding how nurse leaders impact nurses' decisions to leave or stay in their positions. Giorgi's phenomenological method provides a comprehensive analysis to improve the reliability and credibility of the results. Although the study's sample consists of 12 participants, which is adequate for qualitative research focusing on in‐depth experiences, the sample is relatively small. This limited sample size may not be sufficient to represent the broader nursing workforce fully. Another significant limitation is the underrepresentation of female nurses in the sample. With 75% of the participants being male, this does not reflect the general composition of the predominantly female nursing profession. Gender may play a crucial role in determining experiences of communication, support, stress, job satisfaction, professional development and leadership styles within nursing settings. The gender imbalance in the study raises concerns about how this demographic imbalance might affect the applicability of the findings.

A further limitation of this study is the overrepresentation of new graduate nurses, who constituted 50% of the participants. The findings may primarily reflect the experiences and difficulties nurses face in the early stages of their careers. New graduate nurses often experience professional difficulties and unique stressors related to workplace integration, lack of confidence, job training and fear of making mistakes compared to more experienced nurses. These difficulties could influence their decisions to leave their positions. Therefore, orientation programs with clear job descriptions are critical in supporting new nurses during their transition into the workforce (Ernawaty et al. 2024). These programs that provide clear role expectations, mentorship and adequate support systems are essential in enhancing job satisfaction and reducing turnover for new nurses.

One important limitation is the potential impact of geographical mobility. Internationally, many new graduate nurses leave their home countries to pursue career opportunities in other countries that may offer better salaries, professional development opportunities, specialisation options or working conditions. This factor is frequent among new graduate nurses, who may believe their home countries do not provide the specialisation options or career advancement they are seeking. This phenomenon may have impacted the turnover decisions, especially among new graduate nurses who may be more likely to search for new career paths that align with their goals. Another limitation of this study is the lack of information on the turnover rate of the units where each nurse worked. Without information on the turnover rate for each unit, it is difficult to approve whether leadership behaviours are the primary reason to drive nurses away. It might be other factors that drive nurses away. Identifying the turnover rate can impact how significant leadership's role is in the nurses' decisions to leave.

Future research could investigate the impact of different leadership styles on nurse retention. Also, future quantitative studies could provide more rigorous and systematic data on the extent of turnover. Future studies should have a more gender‐balanced study sample. This could provide clearer insight into how leadership styles impact nurses' turnover across gender lines. Furthermore, future research should include a more balanced sample of nurses across different career stages to gain a better understanding of turnover dynamics in nursing.

### 5.2 Implications for Nursing Administration

To reduce nurse turnover, nurse leaders should encourage effective communication between all staff members inside the work setting through open feedback channels, regular meetings and transparent decision‐making processes. Nurse leaders should inform their staff members about any new procedures, organisational updates or policy changes. This allows staff nurses to share feedback, listen well, build trust, build strong relationships, and gain respect. Therefore, this can create a work environment where nurses feel valued and heard. Furthermore, nurse leaders should be trained in open communication and active listening to address the needs and better understanding of their staff.

Additionally, nursing administration must invest in structured leadership development programs, including formal leadership training and mentorship initiatives. Leadership training programs may focus on practices of communication skills, transformational leadership, and conflict resolution. These programs will better prepare nurse leaders to support their teams. Such programs should emphasise empowerment, coaching and professional development, which can reduce turnover rates among staff by increasing job satisfaction and engagement. Furthermore, investing in mentorship programs can make a new or struggling nurse feel more connected to their roles by pairing them with an experienced leader who can guide them, provide practical advice, foster a sense of belonging and provide emotional support. This initiative can help new hires nurses to reduce the feelings of isolation or burnout.

Moreover, nursing administration should maintain stability in the workplace to provide a sense of security to employees. When changes in management positions or policies are necessary, nurse administration should define clearly the purposes and the needs of change, communicate effectively and implement the change gradually and with adequate support to all staff members. This approach would help staff adapt to the changes smoothly, build a reliable and stable workplace setting, and therefore reduce the turnover rate among staff nurses. Nurse administration should also be proactive in making appropriate staffing levels, monitoring staff workload, and advocating for equitable workload distribution, which can help stay and retain staff nurses at the same healthcare organisation for a longer time. Moreover, nursing administrations should create clear job pathways; prioritise training programs and continuous education; provide coaching and mentorship; and offer support for advanced degrees and certifications. These approaches would help the staff to pursue personal growth, remain committed to their positions, and predict their future within the organisation.

## 6 Conclusion

The current study highlights valuable insights into the role of nurse leaders in impacting nurse turnover. This study offers clear evidence that nurse leaders' practices significantly influence nurses' decisions to leave their positions. Poor communication, lack of leader support, unreasonable workloads and staffing issues, inconsistent leadership and policies, and lack of professional growth opportunities have been the main reasons that contribute to nursing turnover. Highlighting these reasons can develop strategies and interventions to create a healthy workplace environment that encourages nurses to stay longer at the same organisation. As a result, productivity, overall morale, quality of patient care, and service will be positively affected inside the healthcare organisation.

## Author Contributions

**Saleem Al‐Rjoub:** made substantial contributions to conception and design, or acquisition of data, or analysis and interpretation of data. **Saleem Al‐Rjoub:** involved in drafting the manuscript or revising it critically for important intellectual content. **Saleem Al‐Rjoub:** given final approval of the version to be published. Each author should have participated sufficiently in the work to take public responsibility for appropriate portions of the content. **Saleem Al‐Rjoub:** agreed to be accountable for all aspects of the work in ensuring that questions related to the accuracy or integrity of any part of the work are appropriately investigated and resolved.

## Ethics Statement

The study was approved by the Institutional Review Board (IRB) of The Hashemite University university on November 19, 2023 (No: 26‐1‐2023‐2024). Informed consent was gained from all participants. The confidentiality of all participants is assured by assigning a number for each participant, which was used to analyse and collect the findings.

## Conflicts of Interest

The author declares no conflicts of interest.

## Data Availability

The data that support the findings of this study are available on request from the corresponding author. The data is not publicly available due to privacy or ethical restrictions.
